# Paracoccidioidomycosis of the larynx: Cases Report

**DOI:** 10.5935/1808-8694.20130141

**Published:** 2015-10-08

**Authors:** Claudiney Candido Costa, Valeriana de Castro Guimarães, Marina Neves Rebouças, Edson Junior de Melo Fernandes

**Affiliations:** a PhD in Medicine - Otolaryngology; Adjunct Professor and Head - Department of Otorhinolaryngology, Medical School, Federal University of Goiás; b PhD in Health Sciences; Speech Epidemiologist. Department of Otorhinolaryngology, Medical School, Federal University of Goiás, Goiânia, GO; c ENT. Otolaryngologist. Medical School, Federal University of Goiás, Goiânia, GO; d MD. Resident Physician, Department of Otorhinolaryngology, Medical School, Federal University of Goiás, Goiânia, GO; Medical School, Federal University of Goiás

**Keywords:** larynx, paracoccidioidomycosis, sulfamethoxazole, trimethoprim-sulfamethoxazole combination

## INTRODUCTION

Paracoccidioidomycosis is a severe systemic disease caused by ***Paracoccidioides brasiliensis*** (P. ***brasiliensis)***. It is a thermal dimorphic fungus, usually acquired through the respiratory tract by inhalation of spores in the air. The infection is insidious and chronic, characterized by the appearance of lesions in the oral and nasal cavities, pharynx, larynx, gums, tongue, soft palate, adrenal glands, liver, bones, gastrointestinal tract, lungs, skin, lymph nodes and nervous system. Dysphonia, dyspnoea, sore throat, dysphagia, weight loss, fever and cough may present as the initial symptoms of the disease. Men over 40, smokers and/or alcohol drinkers are more affected1., 2., 3..

In this report, the authors describe three patients with laryngeal paracoccidioidomycosis treated at a public hospital in the Midwest of Brazil.

## CASE REPORT

### Case 1

**Figure 1:**
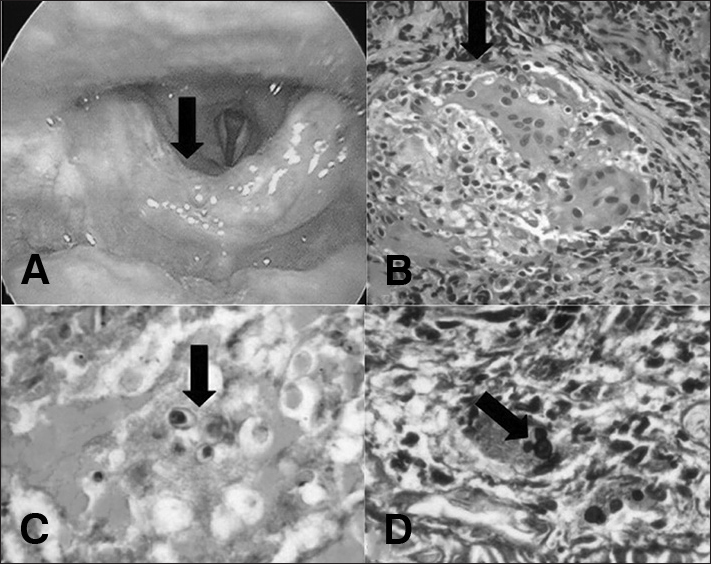
Laryngoscopy: Ulcerative-infiltrative-vegetative lesion on the anterior surface of the epiglottis (a); Histopathology: HE 200x granuloma (b); *fungus Paracoccidioides* PAS 100x (c); Grocott 400x Mickey-Mouse-type budding (d).

Male, 56 years old, smoker for 40 years and ex-alcoholic, coming from Aragarças, TO, with an epiglottis lesion. Upon laryngoscopy there was an ulcerative-infiltrative-vegetative lesion in the anterior face of the epiglottis, with cartilage fixation (Figure 1A). The CT scan showed nodules with cavitations in the pulmonary apex compatible with a chronic granulomatous process. The biopsy was performed in an outpatient basis.

### Case 2

Male, 60 years old, smoker, born and living in Santa Rita do Araguaia, GO, complaining of dysphonia, severe dyspnea and weight loss for 3 months. The Videolaryngoscopy revealed an ulcerative-vegetative-infiltrating lesion on the right vocal fold and right-side ventricular band, extending to the entire posterior commissure, fixating the vocal folds. This patient was submitted to an emergency tracheotomy. A larynx biopsy was carried out under general anesthesia.

### Case 3

Male, 60 years, smoker, coming from Goiânia, GO, complaining of dysphonia for six months worsened in recent months. Upon videolaryngoscopy, there was an ulcerative-infiltrative-vegetative lesion on the vocal folds, partially fixating them. Biopsy was performed under general anesthesia. With no clinical improvement, we investigated tracheal stenosis, which was confirmed by bronchoscopy.

In all three cases, histopathological examination identified granulomatous process with fungi, suggestive of ***P. brasiliensis*** (Figures 1B-1D), being treated with trimethoprim-sulfamethoxazole.w

## DISCUSSION

Diagnosis is based on clinical findings and the identification of ***P. brasiliensis***, present in the pathological exam of the lesions1., 2., 4.. In the cases described, the patients had lesions in the larynx, and were submitted to core biopsy, with a histopathology result suggestive of infection by ***P. brasiliensis;***1., 3., 5. Lesions found in cases of paracoccidioidomycosis are similar to laryngeal neoplasia, requiring differential diagnosis, and thus the established approach is the histopathological exam^4^.

Rural workers are at a higher risk because the disease affects mostly individuals whom, by their occupations, are in constant contact with vegetables and earth2., 3.. Corroborating with the literature, in the reported cases the patients are coming from endemic regions for ***P. brasiliensis***, they are males, smokers and ex-alcoholic and two worked with farming.

Machado Filho et al.^3^, evaluated 104 individuals diagnosed with the disease. Of these, approximately 40 % had laryngeal lesions, and the vocal cords and epiglottis were the most affected structures.

The literature describes lung nodules as the most common radiological findings2., 6.. In one of the cases presented, the CT scan showed areas of cavitations with nodules in the lung apex.

In all three cases, the treatment was performed in outpatient clinics with sulfamethoxazole and trimethoprim, due to ease of administration (per os), better compliance and tolerability^6^. Drug maintenance and regular outpatient follow up were followed as suggested by some authors^6^.

## FINAL REMARKS

Paracoccidioidomycosis should be considered in the differential diagnosis of patients with laryngeal lesions, especially those who reside or resided in endemic areas of ***P. brasiliensis***.
